# Evidence of air quality data misreporting in China: An impulse indicator saturation model comparison of local government-reported and U.S. embassy-reported PM_2.5_ concentrations (2015–2017)

**DOI:** 10.1371/journal.pone.0249063

**Published:** 2021-04-21

**Authors:** Jesse S. Turiel, Robert K. Kaufmann

**Affiliations:** 1 Ash Center for Democratic Governance and Innovation, Harvard University John F. Kennedy School of Government, Cambridge, Massachusetts, United States of America; 2 Department of Earth and Environment, Boston University, Boston, Massachusetts, United States of America; Arizona State University, UNITED STATES

## Abstract

This paper analyzes hourly PM_2.5_ measurements from government-controlled and U.S. embassy-controlled monitoring stations in five Chinese cities between January 2015 and June 2017. We compare the two datasets with an impulse indicator saturation technique that identifies hours when the relation between Chinese and U.S. reported data diverges in a statistically significant fashion. These temporary divergences, or impulses, are 1) More frequent than expected by random chance; 2) More positive than expected by random chance; and 3) More likely to occur during hours when air pollution concentrations are high. In other words, relative to U.S.-controlled monitoring stations, government-controlled stations systematically under-report pollution levels when local air quality is poor. These results contrast with the findings of other recent studies, which argue that Chinese air quality data misreporting ended after a series of policy reforms beginning in 2012. Our findings provide evidence that local government misreporting did not end after 2012, but instead continued in a different manner. These results suggest that Chinese air quality data, while still useful, should not be taken entirely at face value.

## 1. Introduction

For several decades, air quality in China has consistently ranked among the world’s worst. Since the beginning of the reform era in 1978, Chinese air pollution has caused tens of millions of deaths [1] and reduced GDP by trillions of dollars [2]. Nonetheless, widespread reporting of real-time air quality data in China began only recently. Today, nearly all Chinese air quality monitoring stations are owned and operated by government officials, which raises questions about the reliability and integrity of the reported data. Because substandard environmental performance can cause local party leaders to be punished and denied promotion, they have a strong incentive to alter the data reported to the public and the central government in a way that understates pollution.

The tendency of Chinese officials to misreport environmental data is widely documented. Government statistics misreport fish catch [3], coal use [4], GDP growth [5], and carbon emissions [6]. Government data also understates the rate at which agricultural land is converted to urban areas [7] and the rate at which burning coal emits carbon dioxide [8]. These misrepresentations make it difficult for the international community to assess Chinese compliance with international treaties [9–11] and prevent domestic policymakers from accurately gauging environmental impacts. For example, government data indicate that urban air quality improved significantly over the past several years [12–14]. While independent data sources confirm this positive trend [15, 16], the magnitude of improvements could be exaggerated if the Chinese government misreports the data.

Chinese central leaders increasingly recognize the dangers posed by inaccurate environmental information, and in recent years government reforms have attempted to improve oversight and increase penalties for officials accused of data fraud. However, government efforts to fix these institutional problems beg the question: are such efforts effective? In this paper, we analyze hourly data for PM_2.5_ from five Chinese cities to evaluate the success/failure of central government efforts to eliminate misreporting of local air quality data.

### 1.1 Bureaucratic incentives for local air quality data misreporting in China

In the absence of democratic elections, all local state and party leaders in China are appointed by government officials at higher levels of the political system. The placement and promotion of these officials is determined by the cadre evaluation system (CES), which ranks the performance of local leaders using a formula that weights various ‘hard’ and ‘soft’ performance metrics. While the CES has traditionally emphasized economic growth, family planning, and maintaining social order, since 2012 the central government has also affirmed environmental performance as an important ‘hard’ target [17]. Each year, environmental targets are passed from central to local party leaders, who then sign a ‘target responsibility document’ with the director of the local environmental protection bureau (EPB). The EPB enforces pollution control measures and collects local pollution data that are then submitted to the central environmental ministry and released to the public [18].

Due to the fragmented nature of China’s political system, city-level EPBs effectively serve two ‘masters,’ each with different policy goals [19, 20]. First, city-level EPBs must achieve the targets set by higher-level environmental bureaucrats, who tend to favor stricter pollution control policies. Second, they must also maintain the support of the city’s Communist Party standing committee, which typically has close ties with local business leaders and tends to favor more growth-oriented policies. In response to such conflicting demands, city-level EPBs use short-term coping strategies to achieve their bureaucratic goals [21]. With bottom-line objectives prioritized above all else, local bureaucrats face immense pressure to report the ‘correct’ numbers to their higher-ups, and some resort to colluding with other local officials or misreporting data [22, 23]. Given these institutional incentives to cheat, official air pollution data in China often is treated with a high degree of skepticism, by both outside observers and the general public.

### 1.2 Evidence of pre-2012 data tampering

In China, concerted attempts to control local-scale air pollution began in 1996 with the adoption of the National Ambient Air Quality Standards (NAAQS). NAAQS mandated the collection and publication of daily data for the atmospheric concentrations of three pollutants: sulphur dioxide (SO_2_), nitrogen dioxide (NO_2_), and suspended particulates with a diameter of 10 microns or less (PM_10_). These three values were aggregated to form a composite measure of general air quality called the air pollution index (API), which ranged from 0 to 500. By the early 2000s, 86 cities across China reported daily API values to the central environmental ministry (then called the State Environmental Protection Administration, or SEPA), which released the data to the public online [24].

At the end of each year, SEPA used API data to rank the performance of cities from best to worst, with the most important metric being the annual percentage of ‘blue sky days’ (days with an average API less than 100). Cities with at least 85% blue sky days were awarded full credit for air pollution control and could be designated as a ‘National Model City for Environmental Protection.’ This designation was intended to spur competition and improve local environmental quality, as city leaders could receive favorable publicity and possibly even increase their odds of promotion [25].

However, the arbitrary dividing line between ‘good’ and ‘bad’ air quality created a strong incentive for local leaders to misreport data when API was close to the blue sky day threshold of 100. Distortions around the blue sky threshold were first noted in 2008 by Andrews [26], who analyzed air quality data from Beijing and found a much higher-than-expected frequency of API values just below 100 (and a correspondingly lower-than-expected frequency just above 100). These distortions were confirmed in a 2012 study by Chen et al. [25], who used daily API data from 37 Chinese cities and found a statistically significant discontinuity at the blue sky threshold of 100. Expanding on this work, Ghanem and Zhang [27] tested air quality data from 86 Chinese cities between 2001 and 2010, finding sharp discontinuities at the blue sky threshold for roughly half the cities in their dataset. Notably, these discontinuities were more pronounced on days with low wind speed and high visibility, which suggests that city officials were more likely to misreport data on days when pollution was harder to visibly detect.

### 1.3 Post-2012 government reforms: The end of local air quality data misreporting?

Beginning in 2012, the central government restructured the country’s air quality monitoring system, in part to discourage local officials from misreporting data [28, 29]. The number of monitored cities was increased from 86 to 363, and API was replaced with the more sophisticated and comprehensive Air Quality Index (AQI), which added ground-level ozone (O_3_), carbon monoxide (CO), and suspended particulates with a diameter of 2.5 microns or less (PM_2.5_). Additionally, after a two-year transition period, all cities were required to report hourly pollution concentrations instead of daily averages, with all data relayed directly from local monitoring stations to the central environmental ministry without any handling by city EPBs. Finally, the blue sky day metric was officially discontinued in 2013. Under the revised Air Pollution Prevention and Control Action Plan (APPCAP), cities were instead judged by their ability to reduce average annual particulate concentrations between 2012 and 2017 [30].

Early reports suggested that these reforms reduced the misreporting of pollution data. Applying Benford’s Law (a statistical benchmark often used to detect financial fraud) to observations between 2008 and 2012, Stoerk [31] found that differences between concentrations of particulate matter reported by government-controlled monitoring stations and the U.S. embassy in downtown Beijing suggested manipulation. However, including observations for 2013 changed the results to suggest that manipulation stopped in that year. Similarly, using data collected between January 2013 and December 2015, Liang et al. [32] showed that hourly concentrations of PM_2.5_ reported by government-controlled monitoring were not lower than concentrations reported at U.S.-controlled stations in five Chinese cities. Together, these results suggest that misreporting of air quality data no longer is an acute problem, at least in China’s largest megacities.

Contrary to these recent findings, we postulate that some government measurements of local air quality still are being misreported. However, the form of misreporting has changed; instead of manipulating around a given threshold, Chinese officials are now more likely to understate pollution during periods when concentrations are high. We test this hypothesis by estimating the relation between hourly PM_2.5_ concentrations measured by U.S. embassies and consulates in five Chinese cities—which we assume to be reported accurately—and concentrations reported by municipal governments in those same five cities. We choose PM_2.5_ because it is the only pollutant measured by stations controlled by both the U.S. and Chinese governments.

## 2. Data and methods

### 2.1 Air quality data

Since January 1, 2015, China’s central environmental ministry (now called the Ministry of Ecology and Environment, or MEE) has published continuous, real-time hourly measurements of PM_2.5_ in 363 Chinese cities. These data are available to the public for 48 hours, after which they are removed from the MEE website. To obtain these deleted data, we use scraped and archived data from Beijing Sinaapp (publicly available at <https://beijingair.sinaapp.com> [33]), which is the only website to preserve continuous hourly measurements of PM_2.5_ concentrations for the five cities in our sample. These data, and all other air quality data used in our paper, are available for public use, and our research complies with the websites’ terms and conditions.

Each city has several local government-controlled monitoring stations. To create a single value for each city that can be compared to the single U.S. station, we create an average hourly value from values reported by individual Chinese stations in each city. This average weights measurements from individual stations based on the inverse of their distance from the station controlled by the U.S. embassy as follows:

$${GOVT}_{t}=\frac{\sum_{i=1}^{n}\left(\frac{z_{it}}{d_{i}}\right)}{\sum_{i=1}^{n}\left(\frac{1}{d_{i}}\right)}$$ (1)

in which z_it_ represents the hourly PM_2.5_ concentration at a given local government-controlled monitoring station, and d_i_ represents that station’s distance (in km) from the city’s U.S. embassy or consulate. This inverse distance weighting (IDL) gives the largest weight to government-controlled stations closest to the U.S. embassy.

To test the degree to which our findings are robust to the weighting scheme used in Eq (1), we repeat the analysis using inverse quadratic distance weighting (i.e. $\frac{1}{d_{t}^{2}}$). The results of this alternative weighting specification, which are described in S1-S3 Tables in S1 File, do not affect our conclusions in a substantive manner.

Observations for hourly PM_2.5_ concentrations at stations operated by U.S. embassies are obtained from the U.S. State Department, and are publicly available at <https://china.usembassy-china.org.cn> [34]. Measurements at U.S. embassies extend through June 30, 2017, which allows us to compare hourly PM_2.5_ concentrations during a 30-month period (January 2015-June 2017) when the two datasets overlap.

As described S4 Table in S1 File, each city contains eight to twelve stations that generally are within 10 km of the station controlled by the U.S. government. The large number of stations per city implies that conditions unique to a single station have relatively little effect on the city average. The importance of local weather conditions is reduced further by the proximity of stations and locations that ‘surround’ the station controlled by the U.S. government.

We also collect hourly PM_2.5_ concentration data from 74 government-controlled monitoring stations in Taiwan, publicly available at <https://airtw.epa.gov.tw/ENG/default.aspx> [35], which we use to test our methodology for Type I errors (Section 3.5).

### 2.2 Statistical methodology

Consistent with our hypothesis that officials report lower values when concentrations are high, we identify the hours when the relation between measurements at embassy-controlled and government-controlled monitoring stations changes in a statistically significant fashion using the following general equation:

$${EMB}_{t}=\alpha_{1}+\beta_{1}{GOVT}_{t}+\beta_{2}{IMP}_{t}+\varepsilon_{t}$$ (2)

in which EMB_t_ is the concentration of PM_2.5_ (μg /m^3^) measured by U.S. embassy-controlled stations during hour t; GOVT_t_ is the corresponding inverse-distance-weighted average concentration measured by local-government controlled stations; IMP_t_ is an impulse that identifies a statistically significant change in this relation at hour t; α, β_1_, and β_2_ are regression coefficients; and *ε*_*t*_ is a random, heteroskedastic regression residual.

In the absence of misreporting, geographical, or meteorological differences between stations, we expect α = 0 and β_1_ = 1.0. These expectations are strongly rejected by estimating the relation across all five cities; ${\hat{\alpha }}_{1}=4.42\;(t=38.1,p<0.000001)$ and $\hat{\beta_{1}}=1.016$ (t = -13.9, p < 0.000001). Alone, these results do not indicate misreporting because measurements can be affected by the instruments used to measure concentrations as well as geographic and meteorological differences between stations. For example, U.S. embassies and consulates are typically located in the urban core of cities, whereas government-controlled monitoring stations are diffused throughout each city, and so embassy-controlled stations are likely to record slightly higher pollution values (e.g. ∝_1_>0 and/or β_1_>1.0). As such, empirical estimates of $\hat{\alpha }\;and\;\hat{\beta }$ alone cannot be used to detect misreporting.

Misreporting could be detected by testing the relation EMB_t_ = *α*_1_+β_1_GOVT_t_+ε_t_ for one or more changes in *α*_1_ and/or β_1_ [36, 37]. However, this approach is not well suited for testing our hypothesis because change points identify systemic changes that continue over an extended period. For example, if one or both of the instruments used to measure PM_2.5_ is not maintained correctly or replaced, the relation between measurements could change for an extended period, and this change would likely alter the intercept *α*_1_. Furthermore, extended changes in *α*_1_ and/or β_1_ are inconsistent with our hypothesis because systematic changes would be easier to detect than changes in individual measurements.

To detect changes for short periods when the value of EMB_t_ is high, we focus on the quantity, sign, and timing of values of *β*_2_ in Eq (1). Values of *β*_2_ that are statistically different from zero identify hours when the relation between measurements reported by Chinese and U.S. controlled stations differs from the relation that prevails during most other hours. This approach is flexible because it contains no *a priori* assumptions about misreporting. Misreporting can occur at any time, be positive or negative, and have any magnitude. These hourly divergences are termed impulses and they can be used to detect misreporting by testing the following hypotheses:

- **Null Hypothesis #1:** The number of impulses is consistent with random chance. Rejecting this null hypothesis would indicate the data are being misreported.
- **Null Hypothesis #2:** The number of negative impulses equals the number of positive impulses. Rejecting this null hypothesis would indicate the data are being misreported in a specific direction.
- **Null Hypothesis #3:** The timing of the impulses is random. Rejecting the third null hypothesis would indicate that data are being misreported during strategically important hours.

To evaluate the degree to which our methodology is robust, we check for Type I and Type II errors by testing hypotheses #4 and #5:

- **Null Hypothesis #4:** Using similar hourly PM_2.5_ concentration data from Taiwan, where no misreporting is suspected, the number of impulses is consistent with random chance. Rejecting this null hypothesis would indicate that the methodology is prone to false positives (Type I errors) and is therefore not well suited to detecting whether the Chinese government misreports data.
- **Null Hypothesis #5:** Using data from two Chinese cities where local officials were caught manipulating data at specific times and locations, the number of impulses during this period is consistent with random chance. Failing to reject this null hypothesis would indicate that the IIS methodology is not well suited to detecting misreporting when and where it is already known to have occurred (Type II error).

The impulses in Eq (2) that are used to test these hypotheses are identified using an econometric technique, impulse indicator saturation (IIS) [38, 39]. IIS creates an impulse (a zero-one dummy variable) for every hourly observation in a sample. Impulses are dropped/retained by (1) dropping irrelevant variables (gauge) based on a specified nominal significance level, (2) retaining relevant variables (potency) near the theoretical average power, based on a significance level that is specified by the user. We specify a significance level of p = .01 because it reduces the likelihood that the impulses are chosen by random chance, but still identifies a sufficient number of observations via random chance that the results can be evaluated statistically (see Eq (3)). The asymptotics of IIS are thoroughly documented [40].

Although developed by statisticians to analyze econometric data, the IIS procedure is used well beyond economics. For example, the IIS procedure is used to identify the timing of the so-called hiatus in climate change [41], and also to identify periods when speculation and policy changes move oil prices away from market fundamentals [42].

To identify significant impulses in our dataset, we use the IIS procedure to estimate Eq (2) using the R-package *gets* (https://cran.r-project.org/web/packages/gets/index.html). Because this software cannot analyze the large number of hourly observations (~20,000) for each city, we break the thirty-month sample into fourteen subsamples, each containing roughly 1,500 hourly observations. Results generated by analyzing these subsamples are not affected by the number of splits, or by choosing unequal splits [43]. Similarly, the IIS methodology is able to analyze both stationary and non-stationary autoregressive processes without bias [44].

All of our tests are based on the null hypothesis that random chance generates the impulses. Using a significance level of p = .01 implies that random chance would identify one impulse for every 100 hourly observations. We test the null hypothesis that the number of impulses retained is not different than the number expected based on random chance with a test developed specifically for the IIS methodology [45]:

$$S_{\mathrm{prop}}=n^{1/2}\left({\tilde{y}}_{c}-\gamma_{c}\right)$$ (3)

in which ${\tilde{y}}_{c}$ is the observed proportion of impulses and γ*c* denotes the gauge (the expected proportion of detected impulses under the null hypothesis of no impulses) in the initial step of the impulse indicator saturation algorithm. The normal approximation to the gauge is first established by selecting the fixed cut-off c to control the frequency of wrongly detected impulses as the sample size n increases. Under the null hypothesis of no impulses in the model, the expected and observed proportion of impulses should be equal and the S_prop_ statistic (with appropriate scaling) follows a standard normal distribution. Rejecting the null hypothesis using the S_prop_ statistic provides evidence that the observed proportion of impulses is significantly different from γ_*c*_ = 0.01, which is the proportion expected by random chance. Rejecting the null hypothesis implies two alternatives; large but infrequent shifts in measuring equipment and/or meteorological conditions, or data misreporting.

We choose between these two possibilities based in part on the signs of ${\hat{\beta }}_{2}$ (Eq (2)). Under the null hypothesis of no misreporting, there is no *a priori* reason to expect more positive or negative impulses. However, if government officials periodically underreport hourly PM_2.5_ concentrations relative to the ‘true’ values measured by U.S. embassies and consulates, we would expect more positive impulses than negative impulses, because a positive impulse identifies an hour when the PM_2.5_ concentration reported by the U.S. embassy is significantly greater (p < 0.01) than implied by the corresponding Chinese station, as given by $\left(\hat{\alpha }+{\hat{\beta }}_{1}{GOVT}_{t}\right)$. We test the null hypothesis that the number of positive and negative impulses are equal with a z-statistic for the proportion of positive impulses (${\beta_{2}}_{t}^{+}={\beta_{2}}_{t}^{-}$) and a t-statistic that the mean value of the impulses (${\bar{\beta }}_{2}$) equals zero ($\frac{{\bar{\beta }}_{2}-0.0}{{se}_{{\bar{\beta }}_{2}}}$).

Finally, we evaluate the null hypothesis that random chance generates the impulses by testing whether impulses are distributed randomly throughout the sample. If local Chinese officials misreport measurements, they are more likely to understate pollution when true PM_2.5_ concentrations are high. Under this alternative hypothesis, we would expect a positive relation between the observed impulses (*β*_2_) and PM_2.5_ concentrations measured at U.S. embassies. We test this third hypothesis by estimating a logistic regression given by Eq (4):

$$Impulse_{t}^{+}=\alpha_{2}+\beta_{3}EMB_{t}+\varepsilon_{t}$$ (4)

in which ${Impulse}_{t}^{+}$ is a binary variable that equals one for hours when β_2_ > 0 (positive impulses). We also estimate a second logistic regression in which the dependent variable equals one for hour(s) when any impulse–either positive or negative–is present (*β*_2_≠0). For Eq (4), positive values of *β*_3_ indicate that high concentrations (as measured by U.S. embassies) increase the likelihood of a positive impulse. This would suggest that Chinese government officials underreport concentrations of PM_2.5_ during periods of heavy pollution, a result which would be consistent with purposeful misreporting. We calculate the threshold (X) at which local officials are likely to understate concentrations as follows:

$$X=exp(\frac{\ln\left(\frac{0.5}{1-0.5}\right)-\alpha_{2}}{\beta_{3}})$$ (5)

where X is the PM_2.5_ concentration at which the likelihood of a positive impulse reaches 50%, and *α*_2_ and *β*_3_ are regression coefficients from the logit model (Eq (4)).

## 3. Results

### 3.1 Null hypothesis #1: Differences between Chinese and U.S. station measures are generated by random chance

Strong evidence for data misreporting is provided by the higher-than-expected frequency of impulses in Eq (2). Using a significance level of 0.01 to identify impulses implies that random chance will generate roughly 1,012 impulses from the 101,245 hourly observations across all five sample cities. However, using this pooled sample to estimate Eq (2) identifies 1,390 impulses, roughly 40% more than expected by random chance. This greater-than-expected number of impulses also is present in four of the five cities (except Shanghai) when tested individually. These results are confirmed statistically by the positive and significant values for the S_prop_ statistic (Section 2.2), which indicate that random chance likely did not generate the large number of impulses estimated from Eq (2) (Table 1). This suggests that the higher-than-expected frequency of observed impulses in four of the five tested cities is caused by non-random weather patterns, changes/poor maintenance of monitoring equipment, and/or purposeful misreporting by local Chinese officials. Two graphical examples of specific 24 and 36-hour time periods with high concentrations of significant impulses (in Beijing and Shenyang respectively) are provided in S1 and S2 Figs in S1 File.

**Table 1:** Test of null hypothesis #1 for the five Chinese cities and pooled model.

Location	Hourly Obs.	Expected Impulses (p = 0.01)	Observed Impulses	Mean Impulse Coefficient (β_2_)	Null Hypothesis #1	
					S_prop_ Statistic	P-Value
**Beijing**	20,843	208	310	18.99	8.33	< 0.001**
**Shenyang**	20,139	201	293	21.23	7.65	< 0.001**
**Shanghai**	19,557	196	189	-2.01	-0.56	0.578
**Guangzhou**	20,292	203	273	16.41	5.83	< 0.001**
**Chengdu**	20,414	204	325	6.403	10.02	< 0.001**
**Pooled**	101,245	1,012	1,390	12.89	14.06	< 0.001**

The test statistic rejects the null hypothesis at p = 0.05 (*) and p = 0.01 (**).

### 3.2 Null hypothesis #2: The number of positive and negative impulses are equal

While a higher-than-expected frequency of impulses can signify a non-random data generation process, the signs associated with *β*_2_ also contain important information about how they are generated. If random chance generates the impulses, the number of positive and negative impulses should be roughly equal. The specification of Eq (2) makes *β*_2_>0 when measurements at stations controlled by the Chinese government are significantly lower than the value implied by the corresponding measurement at the U.S. embassy-controlled station. In the pooled sample (and in four of the five individual cities), more than 63% of the observed impulses are positive. A z-statistic (Table 2) indicates that, for the pooled model and all individual cities except for Shanghai, it is highly unlikely (p < 0.01) that random chance generates the preponderance of positive impulses. This result is confirmed by a two-tailed, one sample t-statistic (Table 2) which evaluates the null hypothesis that the mean coefficient value of significant impulses is zero. Thus, not only are impulses more frequent than expected but, when they do occur, government-controlled stations are far more likely than U.S. embassy-controlled stations to report concentrations lower than the values implied by EMB_t_ = *α*_1_+β_1_GOVT_t_+ε_t_. Although, by itself, this result does not reject the possibility that the high frequency of impulses is generated by weather conditions and/or changes in/poor maintenance of monitoring equipment, the preponderance of positive impulses suggests that the observed divergences are *directional*, which is clearly consistent with the hypothesis that local Chinese officials misreport data in ways that understate population exposure to high concentrations of PM_2.5_.

**Table 2:** Test of null hypothesis #2 for the five Chinese cities and pooled model.

Location	Observed Impulses	% Positive Impulses	Null Hypothesis #2			
			Z-Statistic: IMPt(+) = IMPt(-)	P-Value	T-Statistic: β_2_ = 0	P-Value
**Beijing**	310	63.50%	4.71**	< 0.001**	3.06	0.002**
**Shenyang**	293	78.80%	9.81**	< 0.001**	3.76	< 0.001**
**Shanghai**	189	48.70%	-0.29	0.772	-0.77	0.442
**Guangzhou**	273	67.80%	5.81**	< 0.001**	5.28	< 0.001**
**Chengdu**	325	73.20%	8.32**	< 0.001**	2.72	0.007**
**Pooled**	1,390	67.80%	13.28**	< 0.001**	5.99	< 0.001**

### 3.3 Null hypothesis #3: The timing of positive impulses is random

**Fig 1:**
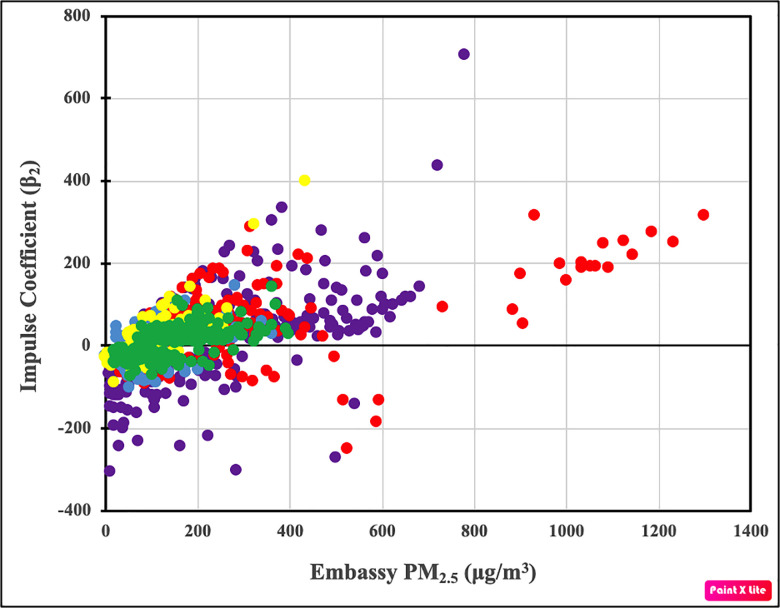
Relation between U.S. embassy-controlled PM_2.5_ concentrations and significant impulses. The coefficient values of all significant impulses were estimated using Eq (2) for Beijing (purple), Shenyang (red), Shanghai (blue), Guangzhou (yellow), and Chengdu (green). Statistical information about these relations is given in Table 3.

If random chance generates the excessive number of positive impulses, they would likely occur randomly throughout the sample. If impulses are generated by non-random weather patterns or changes/poor maintenance of monitoring equipment, the excessive number of positive impulses would likely cluster during periods throughout the sample. By contrast, if the excessive number of positive impulses is generated by Chinese officials purposefully understating measurements, positive impulses are likely to be positively correlated with concentrations measured at U.S. embassies. That is, because officials want to lower annual average pollution concentrations, the greatest incentive to understate pollution occurs during periods when ‘true’ PM_2.5_ concentrations are unusually high. Consistent with this notion, we find a strong positive correlation between observed impulses and hourly PM_2.5_ concentrations measured at U.S. embassies. This positive correlation is evident for the pooled sample (Fig 1), and for each of the five individual cities (Table 3).

**Table 3:** Test of null hypothesis #3 for the five Chinese cities and pooled model.

Location	Null Hypothesis #3					
	Fig 1 r^2^	Logit Model (β_3_)	P-Value (β_3_ = 0)	50% Misreporting Threshold (μg/m^3^)	# Hours > 50% Threshold	# Positive Impulses > 50% Threshold
**Beijing**	0.335	0.011	< 0.001**	565	41	19
**Shenyang**	0.142	0.01	< 0.001**	536	25	18
**Shanghai**	0.218	0.026	< 0.001**	296	2	2
**Guangzhou**	0.551	0.043	< 0.001**	180	59	33
**Chengdu**	0.332	0.021	< 0.001**	350	6	5
**Pooled**	0.289	0.012	< 0.001**	502	133	74

The positive correlation suggested by Fig 1 and Table 3 are confirmed by the results of the logit model (Eq (4)). The positive coefficients (*β*_3_) associated with PM_2.5_ concentrations measured at U.S. stations indicate that higher concentrations of PM_2.5_ increase the likelihood of positive impulses (Table 3). These results are confirmed by a second logit model in which the dependent variable assumes a value of one for every significant impulse, regardless of whether it is positive or negative (S5 Table in S1 File). The PM_2.5_ concentration at which the likelihood of a positive impulse surpasses 50% (which we term the 50% misreporting threshold) is 502 μg/m^3^ in the pooled sample, and ranges from 180 μg/m^3^ to 625 μg/m^3^ for individual cities.

**Fig 2:**
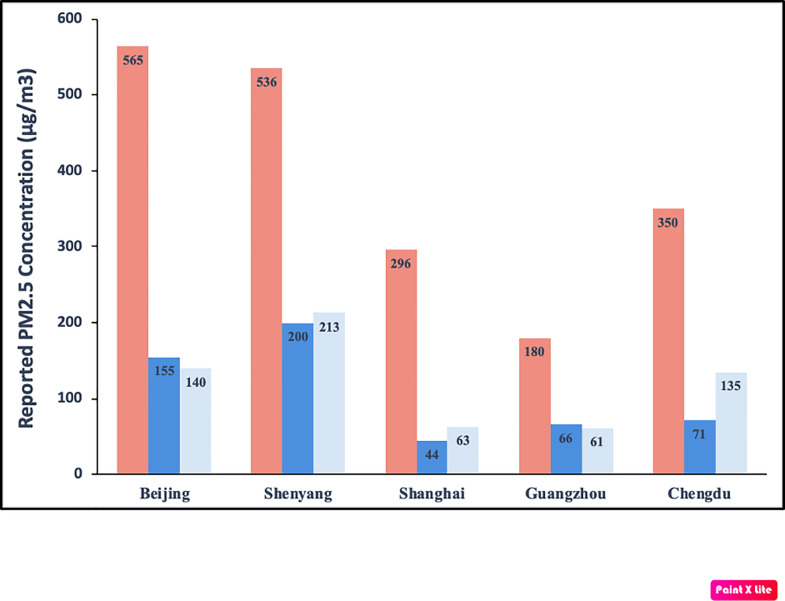
The effect size of positive impulses above the 50% misreporting threshold for each of the five Chinese cities. For each of the five mainland Chinese cities in the dataset, Fig 2 shows the PM_2.5_ threshold at which the likelihood of misreporting exceeds 50% (red). It also shows the average coefficient values for impulses when concentrations at U.S. stations are above this threshold (dark blue), as well as the average difference between Chinese and U.S.-reported PM_2.5_ levels during these impulses (light blue).

To approximate the magnitude of misreporting, we compare average reported PM_2.5_ values between Chinese and U.S.-controlled stations for all positive impulses above the 50% misreporting threshold (Fig 2). The results show that, during hours with significant positive impulses, Chinese-controlled stations underreport U.S.-controlled stations by between 63 μg/m^3^ and 304 μg/m^3^. In percentage terms, Chinese stations underreport U.S. stations by between 18% (Shanghai) and 41% (Guangzhou) during these hours.

Together, the results of hypothesis #3 are consistent with strategic efforts to hide hours with unusually high pollution levels. Conversely, this finding is inconsistent with the ‘clustered pattern’ that would result if the impulses are generated by the poor performance of the instruments used to measure PM_2.5_ and/or local weather.

### 3.4 Robustness checks: Testing the IIS methodology

The impulse indicator saturation technique identifies periods when government-controlled monitoring stations underreport PM_2.5_ concentrations in a systematic, non-random fashion. These results could be caused by local Chinese officials misreporting high concentrations of PM_2.5_ and/or biases in our methodology. To assess the degree which the IIS technique is prone to false positives (Type I errors) or false negatives (Type II errors), we apply the same methodology to samples in which we ‘know’ the ‘correct’ results.

To test for Type I errors, we analyze hourly measurements of air pollution from Taiwan. We assume that Taiwanese officials do not misreport pollution data because Taiwan exhibits greater levels of environmental transparency and because all monitoring stations are controlled directly by the central government. Under these conditions, our methodology should not detect large numbers of positive impulses when concentrations are high. If our methodology detects widespread evidence of data misreporting in Taiwan, this would suggest that our statistical methodology is prone to false positives.

Conversely, we test for Type II errors by analyzing data in cities where the Chinese MEE admits that air quality data were manipulated by local officials at known times and at known locations. For these times and locations, our methodology should detect large numbers of positive impulses when embassy-reported PM_2.5_ concentrations are high. If our methodology does not detect evidence of data misreporting when it is known to have occurred, this would suggest that our statistical methodology is prone to false negatives.

### 3.5 Null hypothesis #4: Measurement errors in Taiwan are caused by random chance

Because U.S. diplomatic outposts in Taiwan do not report air quality data, we test our methodology by choosing the five pairs of Taiwanese monitoring stations (out of 74 total stations) that are separated by the shortest geographical distance (average distance ≈ 4.3km). Although we cannot assume that either of the two government-controlled monitoring stations represent ‘true’ pollution values, as long as both stations don’t misreport air quality data simultaneously and by the same magnitude, any misreporting at either station will still cause a significant impulse.

The null hypothesis of no misreporting implies that our methodology should identify impulses at a frequency approximately equal to the significance level of the criterion used to identify impulses (p = 0.01). Consistent with this notion, our results fail to reject the null hypothesis that the frequency of observed impulses is generated by random chance. None of the S_prop_ statistics in Table 4 are significant at a level (p < 0.05), which suggests that the number of impulses retained by the IIS technique is not significantly greater than the number that would be generated by random chance. This null hypothesis is nearly rejected by Pair 1 (p = 0.069), but in this case there were *fewer* impulses than expected by random chance, rather than more as we would expect with purposeful misreporting.

**Table 4:** Analysis of stations in Taiwan with results generated by applying the IIS methodology to five station pairs.

Station Pairs	Location	Distance (Km)	Hourly Obs.	Expected Impulses (p = 0.01)	Observed Impulses	S_prop_ Statistic	P-Value
**Renwu + Nanzi Districts**	Kaohsuing City	4.8	20,180	202	180	-1.82	0.069
**Qianjin + Zuoying Districts**	Kaohsuing City	4.7	19,652	197	211	1.22	0.221
**Qianzhen + Fuxing Districts**	Kaohsuing City	0.7	20,165	202	196	-0.47	0.637
**Taixi + Mailiao Townships**	Yunlin County	8.3	20,023	200	219	1.57	0.116
**Zhongming + Xitun Districts**	Taichung City	3.2	21,888	219	231	0.97	0.332

These results suggest that the methodology used to analyze concentrations of PM_2.5_ in Chinese cities is not prone to Type 1 errors. As such, false positives probably do not cause us to (incorrectly) conclude that the Chinese government understates concentrations of PM_2.5_ during periods of high concentrations. Furthermore, the failure to reject the null hypothesis using Taiwan data also suggests that errors/changes in instrumentation and/or local variations in weather are not responsible for the preponderance of impulses found in four of the five mainland Chinese cities analyzed.

### 3.6 Null hypothesis #5: Government misreporting causes the large number of positive impulses in Chinese cities

To test whether our methodology correctly identifies instances of data falsification in Chinese cities, we test for Type II errors by analyzing data from cities where local officials admit to underreporting measurements for PM_2.5_. In both Xinyu City of Jiangxi Province and Xinyang City of Henan Province, the local EPB director hired individuals to falsify air quality measurements during September and October of 2017 (the exact dates and hours of tampering were not released by the MEE). According to the MEE, both instances of manipulation involved physical tampering with air quality monitors, including stuffing cotton yarn into sensors and spraying them with mist from ‘fog gun cars’ [46]. More importantly for the test of our methodology, officials manipulated only one monitoring station in each city, leaving all other stations unaffected.

For both Xinyu and Xinyang, we use the IIS methodology to compare hourly PM_2.5_ measurements from the corrupted monitoring station to an average of hourly measurements from the surrounding, non-corrupted monitoring stations. To identify periods when data may be misreported, we break the full sample (January 2016 to December 2018) into eighteen two-month subsamples. If our methodology is not prone to Type II errors, the period when local officials misreported data (the September 2017 –October 2017 subsample) should contain more impulses than expected by random chance. It should also contain more positive than negative impulses.

Consistent with these predictions, test statistics reject the null hypothesis that the number of impulses is consistent with random chance for both cities in the September 2017 –October 2017 subsample (Table 5). Similarly, test statistics reject the null hypothesis that the mean value of impulses is equal to zero. Instead, the number of positive impulses is significantly higher than expected in both cities.

**Table 5:** Results for stations in China where local officials were caught misreporting data.

Subsample Time Period	Xinyu City (Jiangxi Province)						Xinyang City (Henan Province)						
	Hr. Obs.	Obs. Imp.	S_prop_ Stat.	Mean Imp. Coef. (β_2_)	T-Stat: β_2_ = 0	P-Val	Hr. Obs.	Obs. Imp.	S_prop_ Stat.		Mean Imp. Coef. (β_2_)	T-Stat: β_2_ = 0	P-Val
**Jan 2016—Feb 2016**	7,018	84	1.95	0.202	1.60	0.110	5,626	88		5.01**	0.433	4.84	< 0.001**
**Mar 2016—Apr 2016**	7,187	101	4.07**	0.313	3.76	0.001**	5,768	63		0.83	0.212	0.64	0.523
**May 2016—Jun 2016**	7,226	59	-1.85	-0.045	1.87	0.062	5,644	60		0.56	0.015	0.41	0.682
**Jul 2016—Aug 2016**	7,215	72	-0.02	0.084	0.01	0.994	5,803	67		1.39	0.133	1.12	0.264
**Sep 2016—Oct 2016**	7,170	65	-0.94	0.044	0.74	0.462	5,760	62		0.69	0.059	0.52	0.604
**Nov 2016—Dec 2016**	7,155	83	1.6	0.151	1.30	0.193	5,714	50		-1.12	-0.034	0.88	0.377
**Jan 2017—Feb 2017**	7,020	95	3.51**	0.292	2.88	0.004**	5,616	71		2.35*	0.248	1.98	0.048*
**Mar 2017—Apr 2017**	7,280	91	2.53*	0.214	2.07	0.038*	5,824	75		2.60**	0.316	2.15	0.032*
**May 2017—Jun 2017**	7,150	64	-1.05	-0.057	0.84	0.405	5,720	60		0.44	-0.111	0.51	0.610
**Jul 2017—Aug 2017**	7,235	87	2.04*	0.122	1.67	0.095	5,788	52		-0.92	-0.089	0.72	0.473
**Sep 2017—Oct 2017**	7,110	106	4.90**	0.276	4.31	< 0.001**	5,688	93		5.67**	0.49	5.21	< 0.001**
**Nov 2017—Dec 2017**	7,208	81	1.25	-0.119	1.00	0.319	5,624	68		1.86^+^	0.188	1.77	0.076
**Jan 2018—Feb 2018**	7,076	75	0.6	-0.061	0.45	0.655	5,585	55		-0.13	-0.12	0.05	0.962
**Mar 2018—Apr 2018**	7,250	68	-0.63	0.111	0.47	0.636	5,704	51		-0.95	-0.02	0.74	0.460
**May 2018—Jun 2018**	7,111	66	-0.72	0.09	0.55	0.583	5,716	48		-1.44	-0.106	1.15	0.249
**Jul 2018—Aug 2018**	7,198	63	-1.25	-0.025	1.01	0.315	5,775	62		0.66	0.189	0.50	0.620
**Sep 2018—Oct 2018**	7,220	86	1.92	0.137	1.61	0.108	5,604	61		0.78	0.105	0.63	0.529
**Nov 2018—Dec 2018**	7,153	75	0.49	-0.05	0.35	0.724	5,648	64		1.19	-0.083	0.94	0.348

In Table 5, the row containing the subsample period (September 2017 –October 2017) where data misreporting was known to have occurred is highlighted in yellow.

Furthermore, the test of our methodology expands our understanding of what happened in Xinyu and Xinyang. None of the subsamples after October 2017 reject either of the two null hypotheses at a p < 0.05 significance level, which suggests that local officials stopped misreporting data after being caught and punished by the central government. Conversely, both null hypotheses are rejected in some of the subsamples before September 2017 (such as January 2015 –February 2015 in Xinyang city and January 2016 –February 2016 in Xinyu City). Together, these results suggest that local officials in Xinyu and Xinyang were misreporting air quality data well before the period in which they were caught. Finally, the small number of impulses observed in the periods after misreporting was detected also suggests that errors/changes in instrumentation and/or local variations in weather are not responsible for causing the high frequency of impulses present in our main dataset.

## 4. Discussion

Our results strongly suggest that some local Chinese officials continued to misreport measurements of PM_2.5_ concentrations in many of the country’s largest megacities, even after the government’s post-2012 policy reforms. Consistent with our findings, in early 2018 the MEE announced that it had caught officials in seven cities manipulating data during the previous year [46]. Moreover, in April 2018, the central Ministry of Public Security (MPS) charged sixteen local officials in Linyi, Shanxi Province, with tampering with air quality monitors fifty-three times between April 2017 and March 2018 [47].

Our findings of ongoing air quality data misreporting in China are not surprising, because the government’s post-2012 reforms did not eliminate incentives for local officials to cheat. Although requiring hourly, real-time measurements and abolishing the blue sky day metric eliminated manipulation around a given API threshold, local EPBs still face enormous pressure to report pollutant concentrations that decline continuously year-over-year. This pressure is compounded by the fact that the central government has increased penalties for local cadres in failing cities without also increasing the flow of centrally-backed resources or financial support [48]. Thus, faced with increasingly difficult attainment targets and a persistent lack of resources, some local officials have taken the path of least resistance by continuing to misreport air quality data.

Nonetheless, the persistence of local data misreporting does not invalidate results which that suggest urban air quality in China has improved in recent years [12–16]. Even measurements from U.S. embassies and consulates show that annual concentrations of PM_2.5_ fell by more than 25% between 2013 and 2017. Although these broader trends are clear, day-to-day air quality numbers remain highly suspect, especially on high pollution days. The fact that air pollution data is less likely to be accurate on highly polluted days is of particular importance, because acute health effects appear to be more strongly related to hourly peak concentrations than daily averages [49]. Moreover, even though nationwide concentrations of certain pollutants are likely decreasing, local data misreporting makes it difficult for central officials to determine which cities are driving these improvements and which are free-riding.

Central leaders are aware of this problem, but until recently their policy responses have been slow and ineffectual. Starting in late 2016, the central government instituted a series of new reforms aimed at improving environmental governance. Specifically, Beijing announced a transition towards a more centralized environmental bureaucracy that seeks to reduce the negative impacts of ‘local protectionism’ in environmental management [50]. Also, in September 2016, the power to nominate city-level EPB directors was transferred from city governments to provincial EPBs (although city approval is still required to confirm nominees). Also, at the end of 2020, provincial EPBs (rather than city EPBs) assumed full responsibility for funding and personnel decisions at local monitoring stations [51]. The central government also is increasing its supervision and oversight of local officials, and in July 2017, the MEE began surprise environmental inspections in the Beijing-Tianjin-Hebei region [52]. The party-controlled *People’s Daily* announced that these inspections would become the ‘new normal,’ and by the end of 2017, central agencies had disciplined nearly 12,000 local officials and assessed more than $130 million in fines [53]. These MEE inspections have been carried out in tandem with the Central Organization Department (COD) and the Central Commission for Discipline Inspection (CCDI), which are the two most important groups in determining the promotion prospects of local officials. Together, these most recent reforms may represent the beginning of an effective strategy to combat misreporting of local air quality. However, we cannot fully evaluate these reforms because they do not begin until late-2016 and 2017, and our sample only runs through June 2017. Under these conditions, further research is needed to determine whether these latest reforms have been more successful in curtailing the misreporting of local pollution data.

Overall, China’s post-2012 environmental policy reforms improved the availability of air quality data and helped to reduce pollutant concentrations in urban areas, but they did not dissuade some local officials from misreporting data. Although previous studies erroneously suggest that data misreporting ended after 2012, this paper indicates that it continued; just in different, more difficult-to-detect forms. As the central government continues to implement new efforts to address data fraud, it remains to be seen whether these efforts will be effective or whether they will simply induce local officials to find newer and ever-more-innovative ways to falsify official statistics.

## Supporting information

S1 File (DOCX)
