# Supplementary material for: Evidence of air quality data misreporting in China: An impulse indicator saturation model comparison of local government-reported and U.S. embassy-reported PM2.5 concentrations (2015–2017)
Source: PLoS One. 2021 Apr 21;16(4):e0249063. doi: 10.1371/journal.pone.0249063 (PMC8059859; doi:10.1371/journal.pone.0249063)
Supplement: S1 File — (DOCX) [file pone.0249063.s001.docx]

**Evidence of continued air quality data misreporting in China: An impulse indicator saturation model comparison of local government-reported and U.S. embassy-reported PM_2.5_ concentrations**

Jesse S. Turiel^1*¶^, Robert K. Kaufmann^2¶^

^1^Ash Center for Democratic Governance and Innovation, Harvard John F. Kennedy School of Government, Cambridge, Massachusetts, United States of America

^2^Department of Earth and Environment, Boston University, Boston, Massachusetts, United States of America

* Corresponding author E-mail: jsturiel@hks.harvard.edu

**This file includes:**

S1 to S5 Tables

S1 and S2 Figures

S1-S3 Tables reproduce the results of Tables 1-3 in the main text, with the sole difference that they use inverse quadratic distance weighting (rather than inverse distance weighting) to calculate the hourly government-reported average PM_2.5_ concentrations in each city as described in equation (1). The purpose of this alternative weighting specification is to determine the degree to which our results are robust to different measures of calculating city-wide average pollution concentrations.

*Inverse Quadratic Distance Weighed form of Equation (1):*

$${GOVT}_{t}=\frac{\sum_{i=1}^{n} (\frac{z_{it}}{d_{t}^{2}})}{\sum_{i=1}^{n} (\frac{1}{d_{t}^{2}})}$$

S1 Table. Test of null hypothesis #1 for the five Chinese cities and pooled model (Inverse quadratic distance weighting)

| **Location** | **Hourly Obs.** | **Expected Impulses (p = 0.01)** | **Observed Impulses** | **Mean Impulse Coefficient (β_2_)** | **Null Hypothesis #1** | |
| --- | --- | --- | --- | --- | --- | --- |
|  |  |  |  |  | **S_prop_ Statistic** | **P-Value** |
| **Beijing** | 20,843 | 208 | 275 | 16.21 | 6.45 | < 0.001^**^ |
| **Shenyang** | 20,139 | 201 | 292 | 20.90 | 7.65 | < 0.001^**^ |
| **Shanghai** | 19,557 | 196 | 199 | 2.87 | 0.84 | 0.454 |
| **Guangzhou** | 20,292 | 203 | 270 | 15.11 | 5.67 | < 0.001^**^ |
| **Chengdu** | 20,414 | 204 | 311 | 6.04 | 8.82 | < 0.001^**^ |
| **Pooled** | 101,245 | 1,012 | 1,347 | 12.69 | 13.67 | < 0.001^**^ |

S2 Table. Test of null hypothesis #2 for the five Chinese cities and pooled model (Inverse quadratic distance weighting)

| **Location** | **Observed Impulses** | **% Positive Impulses** | **Null Hypothesis #2** | | | |
| --- | --- | --- | --- | --- | --- | --- |
|  |  |  | **Z-Statistic: IMPt(+) = IMPt(-)** | **P-Value** | **T-Statistic: β_2_ = 0** | **P-Value** |
| **Beijing** | 275 | 62.20% | 4.51^**^ | < 0.001^**^ | 2.93 | 0.004^**^ |
| **Shenyang** | 292 | 80.40% | 10.54^**^ | < 0.001^**^ | 3.55 | < 0.001^**^ |
| **Shanghai** | 199 | 46.50% | -0.61 | 0.542 | 0.71 | 0.479 |
| **Guangzhou** | 270 | 67.50% | 5.69^**^ | < 0.001^**^ | 5.05 | < 0.001^**^ |
| **Chengdu** | 311 | 73.90% | 8.44^**^ | < 0.001^**^ | 2.56 | 0.011^*^ |
| **Pooled** | 1,347 | 67.60% | 13.01^**^ | < 0.001^**^ | 6.11 | < 0.001^**^ |

S3 Table. Test of null hypothesis #3 for the five Chinese cities and pooled model (Inverse quadratic distance weighting)

| **Location** | **Null Hypothesis #3** | | | |
| --- | --- | --- | --- | --- |
|  | **Fig 1 r^2^** | **Logit Model (β_3_)** | **P-Value (β_3_ = 0)** | **50% Misreporting Threshold (μg/m^3^)** |
| **Beijing** | 0.297 | 0.01 | < 0.001^**^ | 572 (0.17%) |
| **Shenyang** | 0.161 | 0.01 | < 0.001^**^ | 515 (0.12%) |
| **Shanghai** | 0.177 | 0.022 | < 0.001^**^ | 300 (0.01%) |
| **Guangzhou** | 0.535 | 0.041 | < 0.001^**^ | 181 (0.25%) |
| **Chengdu** | 0.340 | 0.022 | < 0.001^**^ | 339 (0.04%) |
| **Pooled** | 0.284 | 0.011 | < 0.001^**^ | 505 (0.10%) |

The results of S1-S3 Tables show that using inverse quadratic distance weighting does not substantially affect the paper’s main conclusions. In fact, all of the significant p-values found in the original analysis (Tables 1-3 in the main text) remain significant when using inverse quadratic distance weighting.

S4 Table. Distance of government-controlled monitoring stations from the U.S. embassy or consulate in each city.

| **Location** | **# of Government-Controlled Stations** | **Average Distance from U.S. Embassy or Consulate (Km)** | **Median Distance from U.S. Embassy or Consulate (Km)** |
| --- | --- | --- | --- |
| **Beijing** | 12 | 12.2 | 8.2 |
| **Shenyang** | 11 | 5.9 | 5.2 |
| **Shanghai** | 9 | 8.4 | 6.3 |
| **Guangzhou** | 11 | 10.4 | 5.5 |
| **Chengdu** | 8 | 8.9 | 3.9 |
| **Pooled** | 51 | 9.3 | 5.7 |

**S5 Table.** **Logistic regression testing all impulse coefficients (both positive and negative).**

| **Location** | **Logit Model (β_3_)** | **Standard Error** | **P-Value (β_3_ = 0)** | **Pseudo R^2^** | **50% Manipulation Threshold (ug/m^3^)** |
| --- | --- | --- | --- | --- | --- |
| **Beijing** | 0.009 | 0.0004 | <0.001** | 0.17 | 584 (0.14%) |
| **Shenyang** | 0.014 | 0.0006 | <0.001** | 0.25 | 412 (0.01%) |
| **Shanghai** | 0.199 | 0.001 | <0.001** | 0.112 | 291 (0.11%) |
| **Guangzhou** | 0.036 | 0.0014 | <0.001** | 0.27 | 184 (0.19%) |
| **Chengdu** | 0.017 | 0.0009 | <0.001** | 0.116 | 376 (0.22%) |
| **Pooled** | 0.011 | 0.0002 | <0.001** | 0.14 | 485 (0.15%) |

S5 Table is similar to the logistic regression used in the main text (Table 3), but here the binary dependent variable assumes a value of one for all significant hourly impulse coefficients (rather than just positive ones). Significant p-values indicate that impulses are more likely to occur when embassy-measured PM_2.5_ concentrations are high. S5 Table rejects the null hypothesis at p = 0.05 (*) and p = 0.01 (**). Values in parentheses indicate the percentage of days in each sample that exceed the 50% manipulation threshold.

**S1 Fig.** **Example of significant positive impulses (Beijing December 20-21, 2015).**

S1 Fig shows a clear example of a temporary divergence between government-reported (blue) and U.S. embassy-reported (red) PM_2.5_ levels in Beijing covering the 24-hour period beginning at 12:00 on December 20, 2015. During the evening, there are five consecutive hours with significant positive impulses, which are highlighted in yellow.

**S2 Fig.** **Example of significant positive impulses (Shenyang November 8-9, 2015)**

S2 Fig shows another example of a temporary divergence between government-reported (blue) and U.S. embassy-reported (red) PM_2.5_ levels, this time covering a 36-hour period in Shenyang beginning at 0:00 on November 8, 2015 and ending the following day at 12:00. During the day and continuing through the evening on November 8, there are 14 consecutive hours with significant positive impulses, which are highlighted in yellow.
